# Spontaneous motor tempo contributes to preferred music tempo regardless of music familiarity

**DOI:** 10.3389/fpsyg.2022.952488

**Published:** 2022-11-17

**Authors:** Kyoko Hine, Koki Abe, Yuya Kinzuka, Mohammad Shehata, Katsunobu Hatano, Toshie Matsui, Shigeki Nakauchi

**Affiliations:** ^1^Department of Computer Science and Engineering, Toyohashi University of Technology, Toyohashi, Japan; ^2^Division of Biology and Biological Engineering, California Institute of Technology, Pasadena, CA, United States

**Keywords:** tempo preference, spontaneous motor tempo, external music components, familiarity, tapping tempo

## Abstract

Music, and listening to music, has occurred throughout human history. However, it remains unclear why people prefer some types of music over others. To understand why we listen to a certain music, previous studies have focused on preferred tempo. These studies have reported that music components (external), as well as participants’ spontaneous motor tempo (SMT; internal), determine tempo preference. In addition, individual familiarity with a piece of music has been suggested to affect the impact of its components on tempo preference. However, the relationships among participants’ SMT, music components, and music familiarity as well as the influence of these variables on tempo preference have not been investigated. Moreover, the music components that contribute to tempo preference and their dependence on familiarity remain unclear. Here, we investigate how SMT, music components, and music familiarity simultaneously regulate tempo preference as well as which music components interact with familiarity to contribute to tempo preference. A total of 23 participants adjusted the tempo of music pieces according to their preferences and rated the familiarity of the music. In addition, they engaged in finger tapping at their preferred tempo. Music components, such as the original tempo and the number of notes, were also analyzed. Analysis of the collected data with a linear mixed model showed that the preferred tapping tempo of participants contributed to the preferred music tempo, regardless of music familiarity. In contrast, the contributions of music components differed depending on familiarity. These results suggested that tempo preference could be affected by both movement and memory.

## Introduction

Music has been present throughout human history, and music is still enjoyed daily. It has been estimated that an individual listens to as many as 32 h of music per week (Nielsen., 2018). Music is an essential part of our lives, but it remains unclear why humans like to listen to music. To investigate why humans listen to music, studies have explored the influence of individual personality on music preference (Kopacz, 2005; Chamorro-Premuzic and Furnham, 2009; Langmeyer et al., 2012). In addition, recent studies have revealed that music enhances social communication, such as by sharing one’s preferred types of music (Soley and Spelke, 2016; Soley, 2019) as well in terms of individual listening (Lonsdale and North, 2011). Moreover studies of music preference are now applied to enhancing social communication (Kirschner and Tomasello, 2010; Sharda et al., 2018) and regulating individual moods or emotional states (Saarikallio, 2011; Silverman, 2020). Therefore, clarifying why we listen to music should improve our understanding of human diversity, specifically, the differences and commonalities among individuals.

Music consists of certain elements including melody [the succession of pitches (Apel, 2003)]; harmony [the relationship of simultaneous pitches (Apel, 2003)]; rhythm [sound pattern (McAuley, 2010)]; and tempo, which is the interval between successive beats (McAuley, 2010). Tempo is considered the most decisive for emotional expression (Gabrielsson, 2009) and a necessary element that determines the emotional state evoked by listening to music (Hevner, 1937; Rigg, 1964; Gagnon and Peretz, 2003). In particular, music that is played according to a score, such as classical music, is performed more expressively in the performer’s preferred tempo (Repp, 1995). Like performers, listeners have recently been given the ability to change music tempos to their preference using the tempo change function on music player applications. In other words, preferred tempo should contribute to emotional expression while listening to music. Therefore, to clarify why we listen to music, it is important to examine the factors that affect preferred tempo.

Individual differences in tempo preference have been reported (Iwanaga, 1995; Drake et al., 2000a; Karageorghis, 2001; Bauer et al., 2015). Moelants, 2002 collected data regarding performed tempo (rather than perceived tempo) for over 70,000 pieces of music and reported that the preferred tempo of listeners ranged between 67 beats per minute (bpm) and 150 bpm (Moelants, 2002). Moreover, spontaneous motor tempo (SMT) has been suggested to cause individual differences in tempo preference. SMT refers to the pace of mental activity (McAuley, 2010), and it is usually measured by the natural speed of tapping (Fraisse, 1982 for a review) for in-laboratory (e.g., Fraisse, 1982; Collyer et al., 1994; Moelants, 2002) and out-laboratory contexts (Hammerschmidt et al., 2021). Iwanaga (1995) showed that preferred music tempo correlates with individual heart rate, reflecting SMT (Iwanaga, 1995). Additionally, Bauer et al. (2015) conducted an electroencephalographic study and reported that the preferred music tempo was associated with the frequency of motor beta activity recorded during finger tapping (Bauer et al., 2015). These studies demonstrated that preference for performed music tempo is modulated by individual differences in SMT.

While SMT determines the preferred tempo, other studies have shown that music components also determine the preferred tempo. Each piece of music has a specific tempo that is commonly preferred (Iwanaga and Tsukamoto, 1998), indicating that the preferred music tempo is regulated by music components, which are physical parameters of the music, such as an original tempo or pitch. In addition, the distributions of preferred music tempo reportedly change depending on participants’ familiarity with a given piece of music (Iwanaga and Tsukamoto, 1998). Thus, the music components that influence preferred tempo vary according to the familiarity of a piece of music. However, it is unclear which music components are involved in tempo preference and how they are influenced by familiarity.

**FIGURE 1:**
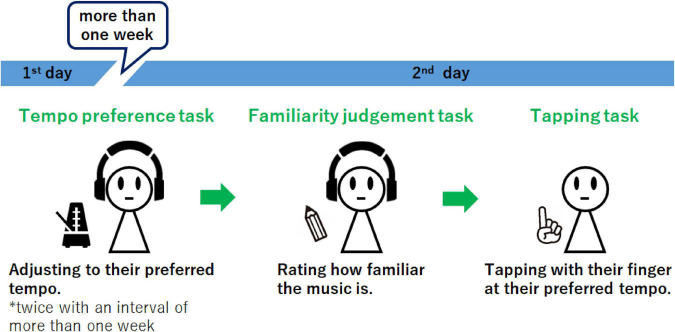
Experimental design. The participants were instructed to set the presented music to their preferred tempo at two time points, separated by more than 1 week. Subsequently, they were instructed to rate the familiarity of each piece of music and completed the tapping task.

As mentioned above, previous studies have shown that SMT and music components are involved in the determination of tempo preference. However, the relationship between SMT and music components in the generation of tempo preference as well as the influence of familiarity have not been investigated. Moreover, the influence of familiarity on music components that contribute to tempo preference for a piece of music has not been clarified. Here, we investigated how SMT and music components simultaneously regulate tempo preference, as influenced by familiarity, and which music components, according to familiarity, contribute to tempo preference. In this study, we conducted a psychological experiment (Figure 1) to clarify the relationships among tempo preference, SMT, music components, and familiarity. In the experiment, the participants were instructed to adjust thirty piano music pieces to their preferred tempo at two time points, separated by more than 1 week, to assess the stability of tempo preference. All pieces of music were presented with the same duration and tempo. Afterward, participants were asked to judge the familiarity of each piece of music. Finally, a tapping task was conducted to assess participants’ SMT. To investigate how SMT, music components, and familiarity simultaneously contributed to the preferred music tempo, linear mixed model analyses were conducted. In these models, the interactions of familiarity (familiar, neutral, unfamiliar) and the preferred tapping tempo as well as each music component (the original tempo, number of notes, event density, pitch, and velocity) were included as fixed effects.

## Materials and methods

### Participants

We calculated sample size using the samplesize_mixed function in R with the sjstats package (Lüdecke and Lüdecke, 2019) with effect size = 0.25 and power = 0.85. A total of 692 sample size, as recommended. We recruited 23 participants (2 females and 21 males, age range: 20 to 25, mean = 22.0, *SD* = 1.3) for this experiment, taking into account the number of musical pieces and the recommended total sample size. All participants were Asian and had normal hearing and normal or corrected-to-normal vision. They all provided informed consent. The experimental procedures were approved by the Committee for Human Research at the Toyohashi University of Technology (approval number: H31-01). All experiments were conducted in accordance with the Declaration of Helsinki.

### Music stimuli

Thirty pieces of music (see Supplementary Appendix Table 1) were selected from the Classical Piano Midi Page, 2018 and mfiles websites (mfiles, 2018). These pieces of music were not recorded from a typical performance by human musicians but were created by inputting the necessary attributions into a MIDI sequencer note for note. All music selections were classical pieces for piano solos, and several experimenters checked there were no changes in tonality or tempo. When one beat was not a quarter note in the MIDI file, the music’s tempo was calculated with a quarter note as a beat. The music’s tempo was between 27 and 200 bpm, in which one beat was defined as a quarter note (crotchet beat on the music sheets). All music was initially presented at 90 bpm for 15 s in the experiment. Therefore, all presented music created at various tempos was presented with the same tempo and duration. Generally, people make judgments that are biased toward an initially presented value (Tversky and Kahneman, 1974). To counteract the influence of the initial tempo as a whole, the initial tempo was 90 bpm, which was approximately equal to the mean of the original tempo (89.7 bpm). These musical data were analyzed with the MIDI Toolbox (Eerola and Toiviainen, 2004) running on MATLAB R2018b (The Mathworks, Natick, MA, USA).

### Procedure

#### Tempo preference task

The participants engaged in a tempo preference task. In this task, the participants were required to adjust the tempo of each piece of music to their preferred tempo. The music was processed with software developed in-house on a personal computer and presented to the participants over headphones (MDR-10, SONY). A computer keyboard was used to obtain the participants’ responses. First, the participants listened to a piece of music for 15 s from beginning of the music. Next, the participants adjusted the music’s tempo to their preferred tempo by pressing the corresponding key (up/down arrow) while listening to the same piece of music. One press of the up arrow increased the speed of the tempo by an increment of 2 bpm; likewise, one press of the down arrow decreased the speed of the tempo by an increment of 2 bpm. Upon a key press, the tempo of the music immediately changed. Once the participants identified their preferred tempo, they pressed the enter key. Until the enter key was pressed, the same music was repeatedly presented. In total, 30 pieces of music were presented to and had their tempos adjusted by the participants. The order of the presentation was randomized. After at least 1 week, the participants performed the tempo preference task once more. The procedure of the task was identical in the first and second instances.

#### Familiarity judgment task

After the second tempo preference task, all participants engaged in a familiarity judgment task. The music presented in the tempo preference task was presented again, at 90 bpm. The participants judged the familiarity of the music on a scale from 1 (extremely unfamiliar) to 7 (extremely familiar) regardless of whether the presented tempo was familiar for each participant. The participants wrote their rating on an answer sheet and then pressed the enter key to advance to the next piece of music. Until the enter key was pressed, the music was repeatedly presented. The participants judged their familiarity with all thirty pieces of music, presented in a randomized order. The familiarity judgment task was conducted after the tempo preference task. Thus, the rating in the familiarity judgment might have been affected by the multiple listening for the participants. Even so, the effect of multiple listenings should occur for all music. Therefore, the rating on the familiarity judgment task was considered to reflect the participants’ familiarity before the experiment.

#### Tapping task

After the familiarity judgment task, a tapping task was conducted. The participants were instructed to tap the index finger of their dominant hand at their preferred tempo. The participants tapped on an iPad screen (Apple) that recorded the tapping rate. During tapping, no stimuli were presented on the screen or speaker. The data were collected in two trials, each with a duration of 30 s. Between the trials, the participants were allowed to take as long of a break as desired. After the tapping task, the participants were debriefed.

## Results

### Music components

All music data were prepared in MIDI files that included the following information on the music components.

#### Tempo

In the current experiment, the tempo specified in the MIDI file is referred to as the original tempo. The music used in this study was constructed from pieces downloaded from websites (Classical Piano Midi Page, 2018; mfiles, 2018) that provide music data recorded in a common performance mode and tempo. The performed tempo of the music used in this study was confirmed to follow the tempo indicated on the scores. The tempo was measured in bpm, defined as the number of beats detected in 1 min. The average original tempo was 89.7 bpm (*SD* = 35.2), and the range was 27–200 bpm.

#### Number of notes

The number of notes represents how many notes are presented in a musical score. The average number of notes was 85.2 (*SD* = 32.0), and the range was 38–156 notes.

#### Event density

Event density is the number of sound events per unit time. The unit of time in the current study was 1 s with 90 bpm. The average event density was 1.57 (*SD* = 0.88). The range was 0.67–5.53.

#### Pitch

The pitch represents how high or low each sound is. A larger number represents a higher-pitched sound. Middle C (C4) has a MIDI pitch number of 60. The average of all pitches presented in this study was 63.3 (*SD* = 5.5). The average score for each piece of music ranged from 54.8 to 80.5.

#### Velocity

The velocity represents how fast the piano key for a note is pressed, which relates to the sound amplitude. The MIDI velocity ranges from 0 to 127. The average velocity of all notes presented in this study was 50.2 (*SD* = 15.7). The average velocity for each piece of music ranged from 32.0 to 97.4.

### Behavioral results

#### Adjusted tempo in the tempo preference task

To check the stability of the adjusted tempo in the tempo preference task over the two trials, the average adjusted tempo in the tempo preference task between the two trials was calculated for each participant. The average adjusted tempos were 99.5 bpm (*SD* = 13.4) on the first day and 99.1 bpm (*SD* = 13.6) on the second day. There was no significant difference between the days [*t*(22) = 0.41, *p* = 0.69, *r* = 0.09]. The correlation between the adjusted tempo on the first and second days was significant (*r* = 0.90, *N* = 23, *p* < 0.001). Moreover, we calculated the correlation between the first and the second preferred tempo for each participant. Then, to estimate a meta-correlation between the adjusted tempo on the first and the second day, we performed a random effects meta-analysis using the R package “metacor.” The meta-correlation between the adjusted tempo on the first and the second day has the mean *r* = 0.57 with a 98% confidence interval (0.46, 0.67) and *p* < 0.001. This implies that the adjusted tempo in the tempo preference task was consistent over time. In addition, the pieces of music were presented in random order on both days. Thus, we calculated the average adjusted tempos on the first and second days for each piece of music for each participant (average = 99.3 bpm, *SD* = 12.7). These values were used as the preferred music tempo in the following analyses. The preferred music tempo ranged from 35 to 224 bpm.

#### Familiarity rating for each piece of music

In the familiarity judgment task, the numbers of music pieces rated as 1 (extremely unfamiliar), 2, 3, 4, 5, 6, and 7 (extremely familiar) were 231, 91, 57, 46, 63, 71, and 131, respectively (Supplementary Appendix Table 2).

#### Tempo in the tapping task

The finger tapping tempo has been reported to be stable over time (McAuley et al., 2006). Thus, the tapping task was not repeated. This task consisted of two trials, each lasting 30 s. The preferred tapping tempo (in bpm) for each participant was calculated as the sum of the number of taps in the two trials. The average preferred tapping tempo was 104.6 bpm (*SD* = 25.3), and it ranged from 54 to 170 bpm (Supplementary Figure 1).

### Effects of the preferred tapping tempo and music components on the preferred music tempo

**FIGURE 2:**
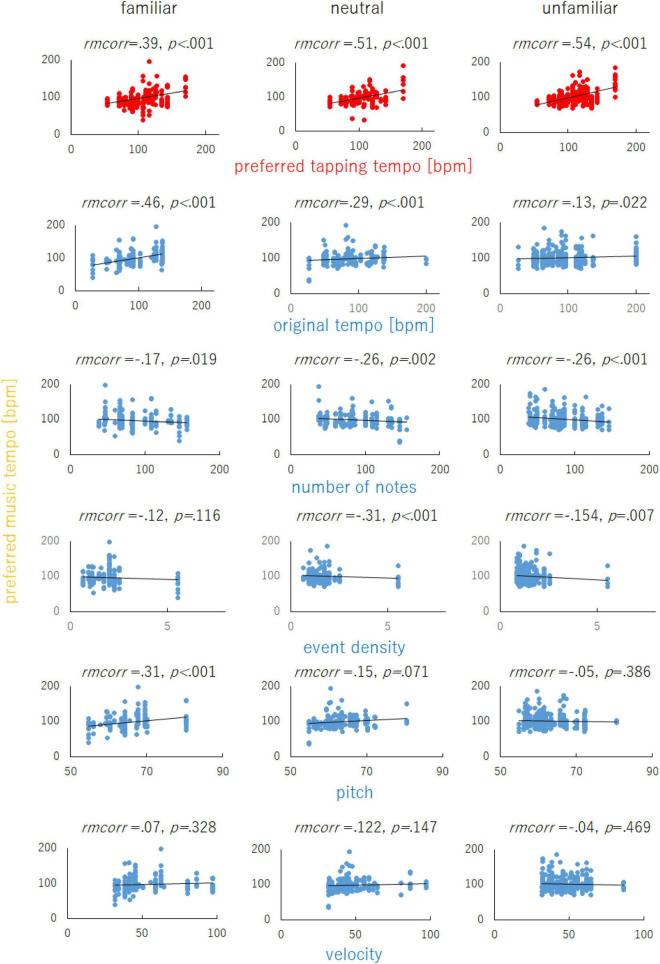
The relationships of the preferred tapping tempo and music components (original tempo, number of notes, event density, pitch, and velocity) with the preferred music tempo for music in the three familiarity categories (familiar, neutral, and unfamiliar). The lines on each graph represent linear fitting curves. Repeated measures correlation (rmcorr) was calculated using the R package “rmcorr”.

All 690 data points (23 participants × 30 music pieces) were divided into three music familiarity categories (familiar, neutral, and unfamiliar) based on participant responses in the familiarity judgment task in order to simplify the results for ease of understanding. The data points rated as 6 or 7 were categorized as familiar (*n* = 202); those rated as 3, 4, or 5 were categorized as neutral (*n* = 166); and those rated as 1 or 2 were categorized as unfamiliar (*n* = 322). Figure 2 shows the relationships of the preferred tapping tempo as well as the music components (original tempo, number of notes, event density, pitch, and velocity) with familiarity category. It is reported that the preferred tapping tempo is usually located between 120 and 130 bpm (Moelants, 2002). Also, it is known that humans often synchronize at rates that are integer multiples or fractions of the basic beat (Parncutt, 1994; Drake et al., 2000b) and prefer tapping tempo that are integer multiples or fractions of 60 bpm (Hammerschmidt et al., 2021). Based on these perspectives, there is a possibility that the fastest (170 bpm) and the slowest preferred tapping tempo (54 bmp) did not reflect the SMT but an integer multiple or fraction of the SMT. Here, we calculated the average of the preferred music tempo for each music, using data excluding data regarding the fastest preferred tapping tempo participant. Then, the average of this value was compared to the average of the preferred music tempo for the fastest preferred tapping tempo participant. The average of the preferred music tempo for the fastest preferred tapping tempo participant (144.1 bpm) was significantly faster than that for the other participants (97.3 bpm) [*t*(29) = 8.94, *p* < 0.001, effect size *r* = 0.86]. Similar to the fastest preferred tapping tempo participant, we calculated the average of the preferred music tempo for each music using data excluding data regarding the slowest preferred tapping tempo participant. The average of the preferred music tempo for the lowest preferred tapping tempo participant (87.8 bpm) was significantly slower than that for the other participants (99.8 bpm) [*t*(29) = 8.50, *p* < 0.001, effect size *r* = 0.85]. These results showed that the fastest/slowest preferred tapping tempo participants preferred the faster/slower music tempo more than the other participants. These results did not support that the observed highest/lowest preferred tapping tempo was an integer multiple or fraction of the SMT. Therefore, no data were excluded from the current analysis.

To assess the contribution of SMT and music components to tempo preference, a linear mixed model was used to test whether the preferred tapping tempo, original tempo, number of notes, event density, mean pitch, and mean velocity predicted the preferred music tempo. To evaluate whether these predictions differed depending on music familiarity, the model included interactions between familiarity and the other predictors (preferred tapping tempo, original tempo, number of notes, event density, pitch, and velocity). The aim of this study was to assess which music components interact with familiarity to contribute to tempo preference, thus, the interaction terms between the familiarity and the preferred taping tempo/the music components were included in the model. Also, the current study was not to aim to clarify which music components significantly contribute to predict the preferred music tempo regardless of familiarity, therefore, the model did not include the main effects. Additionally, the backward elimination of the random effect was analyzed using the step function in R with the lmerTest package (Kuznetsova et al., 2015), which recommended that the music piece and participant ID should be included as random effects. The analysis was performed in R with the lme4 package (Bates et al., 2007) to construct the mixed effects model (Singmann and Kellen, 2019; Brown, 2021).

The linear mixed model analysis showed that AIC = 5758.4, BIC = 5858.2, and conditional and marginal *R*^2^, which were calculated for an *r*^2^ function in R with the performance package (Nakagawa and Schielzeth, 2013), was 0.48 and 0.28, respectively. Additionally, the linear mixed model analyses revealed a significant effect of preferred tapping tempo on the preferred music tempo in all familiarity categories [familiar: *t*(33.63) = 3.77, *p* < 0.001; neutral: *t*(41.16) = 4.47, *p* < 0.001; unfamiliar: *t*(28.78) = 5.27, *p* < 0.001]. A faster preferred tapping tempo predicted a faster preferred music tempo regardless of music familiarity. In addition, there was a significant effect of the original tempo for familiar music [*t*(133.99) = 4.68, *p* < 0.001] but not for neutral [*t*(189.92) = 1.76, *p* = 0.080] or unfamiliar music [*t*(40.23) = 1.44, *p* = 0.159]. For familiar music, a faster original tempo predicted a faster preferred music tempo. The main effect of the number of notes was significant for unfamiliar music [*t*(60.46) =−2.35, *p* = 0.022] but not for familiar music [*t*(64.93) = 0.76, *p* = 0.452] or neutral [*t*(160.50) =−1.17, *p* = 0.245]. There was no significant main effect of event density in any of the familiarity categories [familiar: *t*(71.85) =−1.46, *p* = 0.149; neutral: *t*(113.62) =−1.55, *p* = 0.124; unfamiliar: *t*(100.64) =−0.38, *p* = 0.708]. Similarly, there was no significant main effect of pitch in any of the familiarity categories [familiar: *t*(45.05) =−0.58, *p* = 0.566; neutral: *t*(53.97) = 0.32, *p* = 0.747; unfamiliar: *t*(41.3) = 0.22, *p* = 0.831]. There was no significant main effect of velocity in any of the familiarity categories [familiar: *t*(56.44) =−1.81, *p* = 0.075; neutral: *t*(170.99) =−0.24, *p* = 0.811; unfamiliar: *t*(81.14) = 0.25, *p* = 0.803] (Table 1).

**TABLE 1:** Fixed effects table for the linear mixed model fitted to the preferred music tempo detected in the samples for the familiar, neutral, and unfamiliar music.

	Estimate	SE	df	*t*	*p*	CI (lower)	CI (upper)
Intercept	60.67	13.39	48.75	4.53	<0.001***	38.10	81.62
**Familiar**							
Preferred tapping tempo	0.30	0.08	33.63	3.77	<0.001***	0.16	0.42
Original tempo	0.27	0.06	133.99	4.68	<0.001***	0.17	0.36
Number of notes	0.03	0.05	64.93	0.76	0.452	–0.05	0.12
Event density	–2.37	1.63	71.85	–1.46	0.149	–5.15	0.44
Pitch	–0.11	0.19	45.05	–0.58	0.566	–0.39	0.22
Velocity	–0.17	0.09	56.44	–1.81	0.075	–0.31	–0.01
**Neutral**							
Preferred tapping tempo	0.37	0.08	41.16	4.47	<0.001***	0.24	0.50
Original tempo	0.08	0.05	189.92	1.76	0.080	0.00	0.16
Number of notes	–0.06	0.05	160.50	–1.17	0.245	–0.13	0.03
Event density	–2.61	1.68	113.62	–1.55	0.124	–5.52	0.20
Pitch	0.06	0.19	53.97	0.32	0.747	–0.26	0.38
Velocity	–0.03	0.11	170.99	–0.24	0.811	–0.21	0.15
**Unfamiliar**							
Preferred tapping tempo	0.40	0.08	28.78	5.27	<0.001***	0.27	0.52
Original tempo	0.05	0.03	40.23	1.44	0.159	–0.01	0.10
Number of notes	–0.12	0.05	60.46	–2.35	0.022*	–0.19	–0.04
Event density	–0.69	1.84	100.64	–0.38	0.708	–3.86	2.65
Pitch	0.04	0.19	41.30	0.22	0.831	–0.25	0.36
Velocity	0.03	0.13	81.14	0.25	0.803	–0.18	0.24

*p < 0.05, ***p < 0.001. The 95% confidence intervals (CIs) are shon.

## Discussion

This study aimed to investigate how SMT, music components, and familiarity simultaneously regulate tempo preference and which music components interact with familiarity to contribute to tempo preference. There were two main results.

First, regardless of music familiarity, the preferred tapping tempo, which reflects SMT, significantly predicted the preferred music tempo. Thus, SMT affects tempo preference, regardless of whether a listener is familiar with a piece of music. The internal clock model, in which the internal clock generates time information that affects time perception in various activities, is widely known (Treisman, 1963). Based on this model, a person with a slow internal clock should perceive time quickly, both in the tapping task and the tempo preference task, whereas a person with a fast internal clock should perceive time slowly in both tasks. Time perception affects tempo perception because the tempo is defined as the time interval between events (McAuley, 2010). As a result, individual differences in time perception may significantly correlate with the preferred tapping tempo (in the tapping task) and the preferred music tempo (in the tempo preference task). Another interpretation of these results concerns individual differences in tempo preference rather than tempo perception. People tend to like other individuals who share their values (Morry, 2005, 2007). For example, people prefer others whose features contain a mix their own features to those whose features do not contain their own features (Laeng et al., 2013). In addition, finger tapping tempo is robust across time (McAuley et al., 2006) and correlates with other movement tempos, such as stepping in place (Rose et al., 2020). Therefore, finger tapping tempo could reflect the features of one’s own SMT. If that is the case in the current study, participants might thus prefer music that mixes the original tempo with their own tapping tempo, reflecting the features of their own SMT.

Second, the original tempo predicted the preferred music tempo for familiar music; for unfamiliar music, the number of notes contributed to the prediction. In other words, the music components that affected tempo preference differed depending on music familiarity. The original tempo of the music made a significant contribution to tempo preference only for familiar music. One plausible explanation is that the original tempo for a piece of music is familiar and thus tends to be preferred. Familiarity, which affects our preference, is constructed through exposure to items (Zajonc, 1968), and the number of exposures affects the accuracy of one’s memory (Scarborough et al., 1977). In the current study, original tempo was the tempo indicated on the scores. Typically, music is performed following the tempo indicated on the score, thus, the original tempo should be the same as the typically performed tempo. Some previous studies have shown a significant correlation between the typically performed tempo and the familiarity of the tempo (Halpern, 1988; Levitin and Cook, 1996). These results indicate that the participants may have correctly memorized the original tempo in the current study. If so, the original tempo may contribute to the preferred music tempo because the familiar tempo for a piece of music, which is associated with tempo preference, correlates with the original tempo. Further studies are needed to clarify the relationship among original tempo, familiarity of the tempo, and preferred music tempo. The number of notes significantly contributed to predicting the preferred music tempo for unfamiliar music, whereas the event density did not significantly contribute to predicting the preferred music tempo for any familiarity categories. It could be regarded as that the number of notes, which distinguishes chords and short tones, relates to the quantity of information whereas the event density, which does not distinguish chords and short tones, relates to the temporal resolution of auditory perception. Thus, for unfamiliar music, the amount of information rather than the temporal resolution affected the preferred music tempo in the current study. It is known that time perception and quantity processing are linked to each other (Casini and Macar, 1997; Walsh, 2003 for a review). Additionally, it was found that time and quantity processing share the same cognitive resource (Brown, 1997). If this was the case, when the number of notes was larger, participants might perceive the time under the limited cognitive resources. This might lead them to prefer the slower music tempo because time perception required a longer time under the limited cognitive resources. This interpretation is a speculation. In further studies, the number of notes and the event density as well as the pitch and the velocity should be fully controlled in order to determine how the music components affects the preferred music tempo. Taken together, the findings suggest that tempo preference is determined using the original tempo when a listener has memorized the music; when the listener has not memorized the music, tempo perception (and preference) is determined by the number of notes in the music piece rather than the original tempo.

Moreover, music components influenced the preferred tempo even when participants did not know a music piece (unfamiliar music). For unfamiliar music, the number of notes, which does not directly relate to music tempo, influenced listeners’ tempo preferences. Having shared preferences with others facilitates social communication (Knobloch et al., 2000; Lonsdale and North, 2009; Selfhout et al., 2009; Boer et al., 2011). To share preferences with others, an individual must first determine their own preferences for object features. Thus, when a listener knows a piece of music, the original tempo should lead to a shared tempo preference with others because the original tempo may be known by the listener as well as others. When a listener does not know a piece, they might share the preferences of a group by assessing a specific music component rather than the original tempo. To clarify the role of music in social interactions, the influence of external factors on music preferences must be elucidated.

One might think that the difference in the distribution of the original tempo among the familiarity categories affected the influence of the music component on the preferred tempo. It has been established that initial value (tempo) affects the following judgment (Tversky and Kahneman, 1974). If the original tempo for the familiar music is closer to the initial tempo compared to the original tempo for the neutral and unfamiliar, the initial tempo rather than the original tempo for familiar music may affect the preferred tempo. To determine whether the original tempo for the familiar music was not closer to the initial tempo compared to that for the neutral and the unfamiliar music, we calculated the difference between the original tempo and the initial tempo for all music and assessed whether the difference differed among the familiarity categories. The Shapiro–Wilk test showed that there was no normal distribution for all categories (familiar: *W* = 0.83, *p* < 0.001; neutral: *W* = 0.81, *p* < 0.001; unfamiliar: *W* = 0.90, *p* < 0.001). Thus, the Kruskal–Wallis test was conducted that did not assume data normality for each category. The analysis showed that there was no difference among the familiarity categories (*X_2_* = 2.07, *p* = 0.36) (see also Supplementary Figure 2). In other words, it is not clear that the original tempo for the familiar music was closer to the initial tempo than to the neutral and the unfamiliar music. Therefore, the difference in the distribution of the original tempo among the familiarity categories could not fully account for the current result, and there is still a possibility that the music components that affected tempo preference differed depending on music familiarity.

In the current study, the preferred tapping tempo was collected while participants were seated. The preferred tapping tempo was reported to be faster after running than before running, because the SMT had changed (Dosseville et al., 2002). Based on our results and those of a previous study, the preferred music tempo is thought to be modulated by various situations, for example, during physical activity or just before bedtime. Future research should assess whether the preferred music tempo changes depending on SMT. Another limitation of the current study is that it did not consider participants’ perceptions of other music elements such as the tempo, rhythm, or beat, or the effect of perceived tempo on the preferred music tempo. Therefore, the music tempo perceived by a listener may have affected the preferred music tempo. Actually, there were some music pieces in which the perceived tempo seemed different from the original tempo in the current study (Sonata No. 8 In C minor, Op. 13-2 by Beethoven and Etude No. 6 by Liszt). Additionally, we analyzed the linear mixed model using preferred tapping tempo and original tempo with log scales instead of linear scales (Supplementary Appendix Table 3). Again, similar results were obtained in the additional analysis. It is known that tempo perception or time discrimination follow Weber’s Law (Halpern and Darwin, 1982). Therefore, tempo perception could affect the preferred music tempo. In addition, the rhythm or beat perceived by a listener could influence the perceived tempo (Duke, 1994). Therefore, rhythm or beat might have affected the preferred music tempo in the current study because rhythm, beat, and tempo all affect music preference (Finnä, 1989). Future studies investigating how music preference is determined should consider the interaction among music elements including tempo, rhythm, and beat.

Also, we investigated the relationship between the music components and the preferred music tempo in the current study. Actually, it may be happened that the music components affect the preferred music tempo *via* musical characteristic. However, we did not control and collect the data regarding the musical characteristics in the experiment. Therefore, it could not to clarify how musical characteristics were involved in the preferred music tempo. In further studies, the musical characteristics should be also controlled in experiments, and it must be clarified the role of the musical characteristics on the preferred music tempo.

Moreover, based on the current results, it would be expected that the adjusted preferred music tempo for a piece of music might be driven upward closer to the preferred tapping tempo when the original tempo for the piece is below the preferred tapping tempo for a listener. When the original tempo is faster than the preferred tapping tempo, the adjusted preferred music tempo might be driven downward toward the preferred tapping tempo. Such a relationship between the original tempo and the preferred tapping tempo should be investigated under the condition in which the difference between the original tempo and the preferred tapping tempo is fully controlled.

In addition, participants’ attributions should be considered in future studies. One study has indicated that music experience affected tempo preference (Drake et al., 2000a) while others have insisted that the effect of the music experience was inconclusive (Hammerschmidt et al., 2021). To clarify the involvement of the effect of musical experience, the musicianship for each participant should be assessed and controlled in further studies. Additionally, age and gender should be evaluated with regard to the preferred music tempo. In the current study, all participants were students (age range: 20–25). Older people tend to tap their fingers slower compared to younger people (Hammerschmidt et al., 2021). From this perspective, a broader range of participants is needed in future work to generalize the results obtained in this study. Regarding gender, there were only two female participants in the current study. Since it was reported in a previous study that gender did not affect tempo preference (Karageorghis et al., 2006), it was not expected that gender would affect the current results. Nonetheless, to understand how individual preferred tempo is formulated, it should be assessed how the contribution of SMT and the musical components differ depending on gender, which is a fundamental participant attribution.

The current study showed that SMT affects tempo preference for a music piece regardless of familiarity. Additionally, the original tempo, which might be linked to the memory of a piece of music, contributed to tempo preference only for familiar music. For unfamiliar music, the number of notes contributed to tempo preference. To precisely understand the underpinnings of tempo preference, we should consider both internal and external factors. Recently, music has been increasingly used in the clinical treatment of patients for problems such as dementia, insomnia and depression (Gold et al., 2005; Maratos et al., 2008). For example, listening to slow tempo music has been found to induce a positive mood compared to that induced by fast tempo music (Ooishi et al., 2017). Moreover, listening to preferred music has been shown to reduce anxiety (Walwroth, 2003). However, there are large individual differences regarding whether listening to a piece of music successfully induces a certain mental state (Vink et al., 2003). The results of the current study show that SMT is an internal factor involved in predicting preferred music tempo. In other words, the findings of our study could help in the prediction of personalized preferred tempo, which could contribute to more effective methods of music therapy for individual patients.

## Data availability statement

The raw data supporting the conclusions of this article will be made available by the authors, without undue reservation.

## Ethics statement

The studies involving human participants were reviewed and approved by the Committee for Human Research at the Toyohashi University of Technology. The patients/participants provided their written informed consent to participate in this study.

## Author contributions

KyH, KA, YK, MS, TM, and SN developed the study concept. KA, YK, KaH, and TM prepared the materials. KyH and KA collected and analyzed the behavioral data. KyH wrote the manuscript. All authors discussed the results and commented on the manuscript.
