# Supplementary material for: Spontaneous motor tempo contributes to preferred music tempo regardless of music familiarity
Source: Front Psychol. 2022 Nov 17;13:952488. doi: 10.3389/fpsyg.2022.952488 (PMC9713942; doi:10.3389/fpsyg.2022.952488)
Supplement: Supplementary file 1 [file Table_1.DOCX]

Appendix Table 1. Profiles for 30 pieces of music.

|  | title | composer | original tempo [bpm] | number of notes | event density | pitch (mean) | pitch (median) | pitch (mode) | velocity (mean) | velocity (median) | velocity (mode) | number of bars |
| --- | --- | --- | --- | --- | --- | --- | --- | --- | --- | --- | --- | --- |
| music 1 | Sonata No.8 In C minor, Op.13-2 ‘Pathetique | Beethoven | 27 | 146 | 5.53 | 54.8 | 55.0 | 51.0 | 32.0 | 28.0 | 25.0 | 10 $\frac{1}{8}$ |
| music 2 | Clair de lune | Debussy | 70 | 46 | 1.73 | 70.2 | 70.0 | 70.0 | 34.0 | 34.0 | 36.0 | 5 |
| music 3 | Prelude Op.28-7 | Chopin | 88 | 69 | 1.13 | 64.3 | 66.0 | 73.0 | 58.8 | 61.0 | 61.0 | 9 |
| music 4 | Waltz Op.39-15 | Brahms | 92 | 100 | 1.60 | 61.5 | 65.0 | 72.0 | 59.0 | 57.0 | 51.0 | 8 $\frac{1}{3}$ |
| music 5 | Gymnopedie No.1 | Satie | 82 | 38 | 1.20 | 60.5 | 62.0 | 66.0 | 46.1 | 46.0 | 41.0 | 7 $\frac{1}{2}$ |
| music 6 | Für Elise | Beethoven | 69 | 83 | 2.27 | 64.3 | 69.0 | 76.0 | 38.5 | 38.0 | 38.0 | 13 $\frac{1}{3}$ |
| music 7 | Minuet in G, No.4 from 2nd Notebook | Bach | 128 | 47 | 2.00 | 67.7 | 69.0 | 67.0 | 62.6 | 62.0 | 64.0 | 7 |
| music 8 | Etude No. 6 aus Grandes Etudes de Paganini | Liszt | 137 | 115 | 2.53 | 67.4 | 69.0 | 69.0 | 43.8 | 43.0 | 36.0 | 9 |
| music 9 | La Campanella | Liszt | 92 | 109 | 2.00 | 80.5 | 80.0 | 99.0 | 41.3 | 40.0 | 40.0 | 6 $\frac{5}{6}$ |
| music 10 | Moonlight Sonata | Beethoven | 49 | 82 | 1.73 | 56.1 | 60.0 | 56.0 | 32.3 | 32.0 | 26.0 | 10 $\frac{1}{3}$ |
| music 11 | Marche Militaire | Schubert | 125 | 135 | 0.93 | 59.5 | 61.0 | 57.0 | 86.5 | 92.0 | 96.0 | 9 $\frac{3}{4}$ |
| music 12 | The Entertainer | Joplin | 69 | 156 | 1.00 | 63.7 | 62.0 | 55.0 | 80.5 | 85.5 | 91.0 | 10 $\frac{1}{8}$ |
| music 13 | Pictures at a Promenade I | Mussorgsky | 103 | 67 | 0.67 | 60.5 | 62.0 | 67.0 | 97.4 | 97.0 | 92.0 | 4 |
| music 14 | Klaviersonate KV 545 1.Satz | Mozart | 138 | 67 | 2.00 | 69.6 | 69.0 | 67.0 | 45.3 | 40.0 | 40.0 | 4 $\frac{7}{8}$ |
| music 15 | Träumerei (Reverie) | Schumann | 65 | 60 | 1.20 | 59.7 | 60.0 | 60.0 | 41.9 | 42.0 | 38.0 | 5 $\frac{1}{4}$ |
| music 16 | España, Oups 165 Malagueña | Albeniz | 119 | 78 | 2.27 | 72.1 | 72.0 | 78.0 | 53.5 | 55.0 | 55.0 | 13 $\frac{1}{3}$ |
| music 17 | España, Oups 165 Prelude | Albeniz | 112 | 73 | 1.33 | 72.2 | 69.0 | 64.0 | 51.4 | 51.0 | 48.0 | 7 |
| music 18 | Intermezzo in A major, Op118 No. 2 | Brahms | 49 | 131 | 1.13 | 60.2 | 63.0 | 69.0 | 48.0 | 47.0 | 47.0 | 10 |
| music 19 | The Carnival of the Animals - V. The Elephan | Saint-Saëns | 54 | 136 | 1.00 | 57.9 | 63.0 | 70.0 | 64.9 | 60.0 | 55.0 | 13 $\frac{1}{3}$ |
| music 20 | Petite Suite Nocturne | Borodin | 77 | 69 | 1.33 | 59.1 | 62.0 | 68.0 | 45.9 | 49.0 | 49.0 | 5 $\frac{1}{8}$ |
| music 21 | Petite Suite Rêverie | Borodin | 63 | 80 | 1.07 | 61.8 | 61.5 | 61.0 | 44.8 | 46.0 | 46.0 | 5 $\frac{1}{4}$ |
| music 22 | Prelude No. 23, Opus 28 | Chopin | 116 | 110 | 1.47 | 61.2 | 62.0 | 48.0 | 38.6 | 38.0 | 40.0 | 4 $\frac{1}{16}$ |
| music 23 | Sonatina Opus 36 No. 3, Opus 36 2. movement | Clementi | 90 | 40 | 1.87 | 66.6 | 66.5 | 62.0 | 32.1 | 31.0 | 22.0 | 5 |
| music 24 | Sonatina Opus 36 No. 4, Opus 36 2. movement | Clementi | 51 | 84 | 1.13 | 62.6 | 65.0 | 65.0 | 39.7 | 37.0 | 42.0 | 10 |
| music 25 | Danzas españolas No. 4 Villanesca | Granados | 85 | 83 | 1.47 | 63.4 | 64.0 | 43.0 | 43.9 | 44.0 | 45.0 | 10 |
| music 26 | Danzas españolas No.3 Zarabanda | Granados | 200 | 86 | 1.00 | 57.1 | 59.5 | 62.0 | 61.7 | 60.0 | 58.0 | 7 $\frac{1}{3}$ |
| music 27 | Klaviersonate Hoboken XVI_ 43 2 | Haydn | 120 | 49 | 1.00 | 65.7 | 65.0 | 68.0 | 55.7 | 55.0 | 72.0 | 6 $\frac{1}{6}$ |
| music 28 | Sonate XVI_35 2 | Haydn | 53 | 100 | 1.73 | 63.1 | 65.0 | 70.0 | 50.6 | 51.0 | 60.0 | 5 $\frac{1}{6}$ |
| music 29 | Piano Sonata in A minor, D 784, Opus 143-2 | Schubert | 72 | 70 | 0.87 | 56.6 | 57.0 | 48.0 | 36.8 | 38.0 | 50.0 | 5 |
| music 30 | Der Dichter spricht | Schumann | 96 | 47 | 0.93 | 60.3 | 62.0 | 62.0 | 38.3 | 38.0 | 32.0 | 5 $\frac{1}{4}$ |
