# Supplementary material for: Spontaneous motor tempo contributes to preferred music tempo regardless of music familiarity
Source: Front Psychol. 2022 Nov 17;13:952488. doi: 10.3389/fpsyg.2022.952488 (PMC9713942; doi:10.3389/fpsyg.2022.952488)

Appendix Table 2. The number of data points for each familiarity rate and mean familiarity rating for each piece of music

|  | 1 | 2 | 3 | 4 | 5 | 6 | 7 | mean | SD |
| --- | --- | --- | --- | --- | --- | --- | --- | --- | --- |
| music1 | 3 | 1 | 2 | 2 | 5 | 4 | 6 | 4.78 | 2.02 |
| music2 | 6 | 3 | 0 | 3 | 5 | 3 | 3 | 3.83 | 2.18 |
| music3 | 1 | 1 | 0 | 1 | 1 | 6 | 13 | 6.04 | 1.60 |
| music4 | 9 | 3 | 1 | 3 | 4 | 1 | 2 | 3.04 | 2.07 |
| music5 | 3 | 0 | 1 | 3 | 3 | 5 | 8 | 5.17 | 1.99 |
| music6 | 0 | 0 | 0 | 0 | 0 | 4 | 19 | 6.83 | 0.38 |
| music7 | 0 | 0 | 0 | 1 | 1 | 4 | 17 | 6.61 | 0.77 |
| music8 | 7 | 2 | 2 | 4 | 1 | 4 | 3 | 3.61 | 2.22 |
| music9 | 1 | 1 | 0 | 0 | 4 | 4 | 13 | 6.00 | 1.59 |
| music10 | 5 | 1 | 3 | 3 | 3 | 3 | 5 | 4.17 | 2.20 |
| music11 | 3 | 3 | 4 | 2 | 5 | 3 | 3 | 4.04 | 1.94 |
| music12 | 0 | 0 | 0 | 1 | 1 | 4 | 17 | 6.61 | 0.77 |
| music13 | 0 | 0 | 0 | 3 | 1 | 10 | 9 | 6.09 | 0.97 |
| music14 | 2 | 0 | 3 | 0 | 3 | 8 | 7 | 5.35 | 1.83 |
| music15 | 7 | 4 | 2 | 2 | 5 | 2 | 1 | 3.17 | 1.95 |
| music16 | 17 | 3 | 1 | 0 | 2 | 0 | 0 | 1.57 | 1.17 |
| music17 | 18 | 2 | 1 | 1 | 1 | 0 | 0 | 1.48 | 1.06 |
| music18 | 11 | 4 | 2 | 2 | 2 | 1 | 1 | 2.43 | 1.81 |
| music19 | 10 | 8 | 1 | 2 | 0 | 1 | 1 | 2.17 | 1.61 |
| music20 | 12 | 6 | 1 | 3 | 0 | 0 | 1 | 2.00 | 1.47 |
| music21 | 15 | 2 | 4 | 2 | 0 | 0 | 0 | 1.70 | 1.04 |
| music22 | 12 | 7 | 2 | 1 | 1 | 0 | 0 | 1.78 | 1.06 |
| music23 | 11 | 5 | 3 | 1 | 3 | 0 | 0 | 2.13 | 1.39 |
| music24 | 7 | 7 | 3 | 0 | 6 | 0 | 0 | 2.61 | 1.55 |
| music25 | 8 | 5 | 4 | 1 | 2 | 3 | 0 | 2.70 | 1.76 |
| music26 | 16 | 4 | 2 | 1 | 0 | 0 | 0 | 1.48 | 0.83 |
| music27 | 9 | 4 | 5 | 2 | 2 | 0 | 1 | 2.48 | 1.61 |
| music28 | 7 | 7 | 6 | 0 | 1 | 1 | 1 | 2.48 | 1.58 |
| music29 | 18 | 2 | 2 | 0 | 1 | 0 | 0 | 1.43 | 0.97 |
| music30 | 13 | 6 | 2 | 2 | 0 | 0 | 0 | 1.70 | 0.95 |

Appendix

The mean of the original tempo and confidence interval for each familiarity category. The bottom line shows the original tempo for each music (30 pieces) used in the current study.


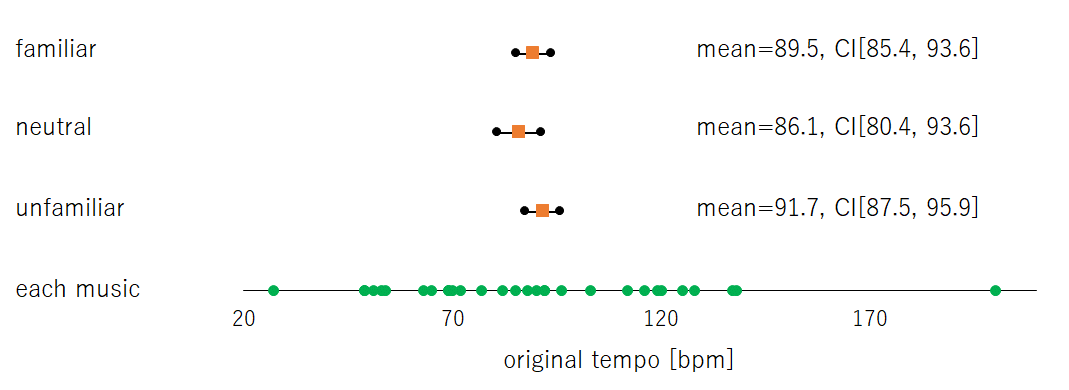

Supplement: Supplementary file 2 [file Table_2.DOCX]
