# Supplementary material for: Spontaneous motor tempo contributes to preferred music tempo regardless of music familiarity
Source: Front Psychol. 2022 Nov 17;13:952488. doi: 10.3389/fpsyg.2022.952488 (PMC9713942; doi:10.3389/fpsyg.2022.952488)
Supplement: Supplementary file 3 [file Table_3.DOCX]

Appendix Table 3. Fixed effects table for the linear mixed model fitted to the preferred music tempo detected in the samples for the familiar, neutral, and unfamiliar music. The 95% confidence intervals (CIs) are shown. Preferred tapping tempo and original tempo were log scales.

|  |  | Estimate | SE | df | *t* | *p* | CI (lower) | CI (upper) |
| --- | --- | --- | --- | --- | --- | --- | --- | --- |
| Intercept | | -92.97 | 38.44 | 29.14 | -2.42 | .022* | -160.22 | -26.44 |
| Familiar | |  |  |  |  |  |  |  |
|  | Preferred tapping tempo (log scale) | 24.63 | 8.19 | 28.05 | 3.01 | <.001*** | 11.40 | 37.49 |
|  | Original tempo (log scale) | 14.60 | 5.39 | 118.70 | 2.71 | <.001*** | 6.29 | 23.81 |
|  | Number of notes | 0.00 | 0.05 | 60.62 | 0.01 | 0.990 | -0.07 | 0.08 |
|  | Event density | 0.20 | 1.77 | 72.35 | 0.11 | 0.910 | -2.60 | 3.26 |
|  | Pitch | 0.21 | 0.29 | 84.55 | 0.71 | 0.480 | -0.30 | 0.67 |
|  | Velocity | -0.02 | 0.11 | 62.88 | -0.21 | 0.833 | -0.21 | 0.14 |
| Neutral | |  |  |  |  |  |  |  |
|  | Preferred tapping tempo (log scale) | 35.62 | 8.19 | 28.13 | 4.35 | <.001*** | 22.77 | 49.38 |
|  | Original tempo (log scale) | 7.62 | 4.25 | 121.80 | 1.79 | 0.076 | 0.00 | 14.40 |
|  | Number of notes | -0.06 | 0.05 | 138.40 | -1.06 | <.001*** | -0.15 | 0.04 |
|  | Event density | -1.87 | 1.74 | 100.60 | -1.08 | 0.284 | -4.62 | 0.99 |
|  | Pitch | 0.06 | 0.29 | 164.30 | 0.20 | 0.844 | -0.43 | 0.59 |
|  | Velocity | -0.02 | 0.12 | 166.40 | -0.21 | 0.837 | -0.23 | 0.16 |
| Unfamiliar | |  |  |  |  |  |  |  |
|  | Preferred tapping tempo (log scale) | 40.89 | 8.04 | 26.11 | 5.08 | <.001*** | 28.48 | 54.06 |
|  | Original tempo (log scale) | 5.78 | 3.25 | 36.38 | 1.78 | 0.083 | 0.33 | 11.18 |
|  | Number of notes | -0.12 | 0.05 | 58.77 | -2.36 | 0.022* | -0.21 | -0.03 |
|  | Event density | 0.05 | 1.88 | 93.38 | 0.03 | 0.980 | -2.96 | 3.32 |
|  | Pitch | -0.19 | 0.23 | 52.76 | -0.80 | 0.426 | -0.64 | 0.23 |
|  | Velocity | 0.00 | 0.13 | 70.81 | -0.02 | 0.983 | -0.24 | 0.23 |
| **p<.05. ***p<.001.* | |  |  |  |  |  |  |  |
