# Supplementary figures and images for: Spontaneous motor tempo contributes to preferred music tempo regardless of music familiarity
Source: Front Psychol. 2022 Nov 17;13:952488. doi: 10.3389/fpsyg.2022.952488 (PMC9713942; doi:10.3389/fpsyg.2022.952488)

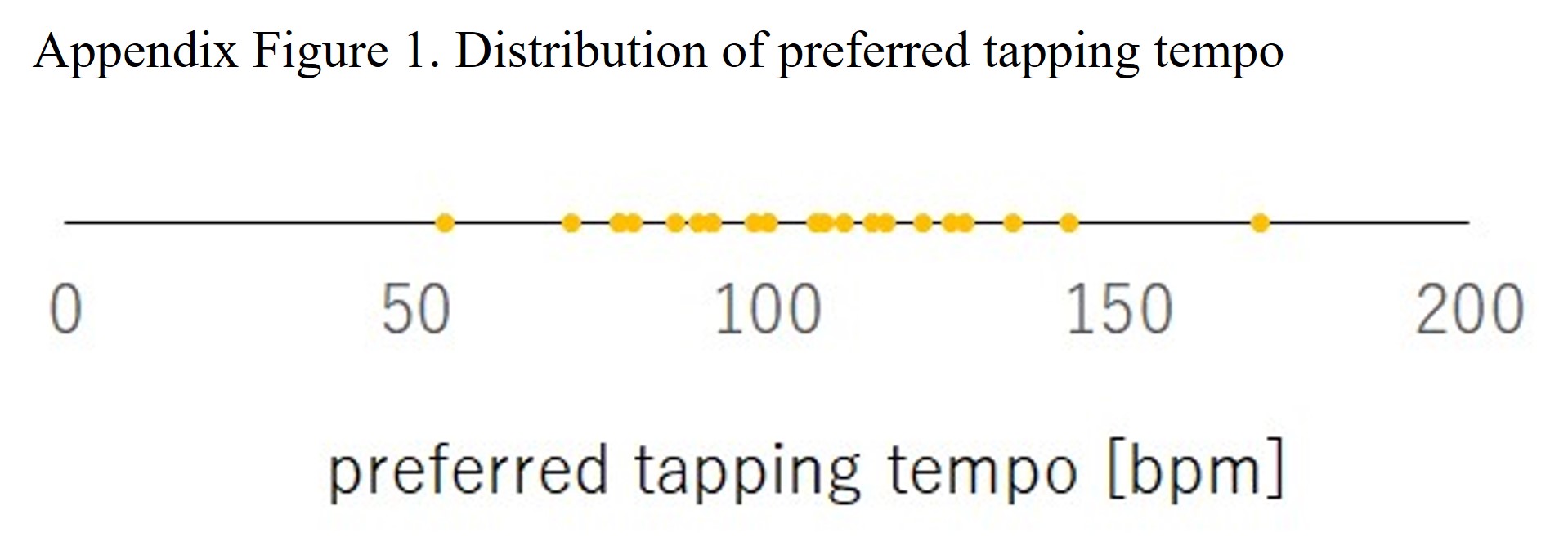

Supplement: Supplementary file 4 [file Image_1.jpg]

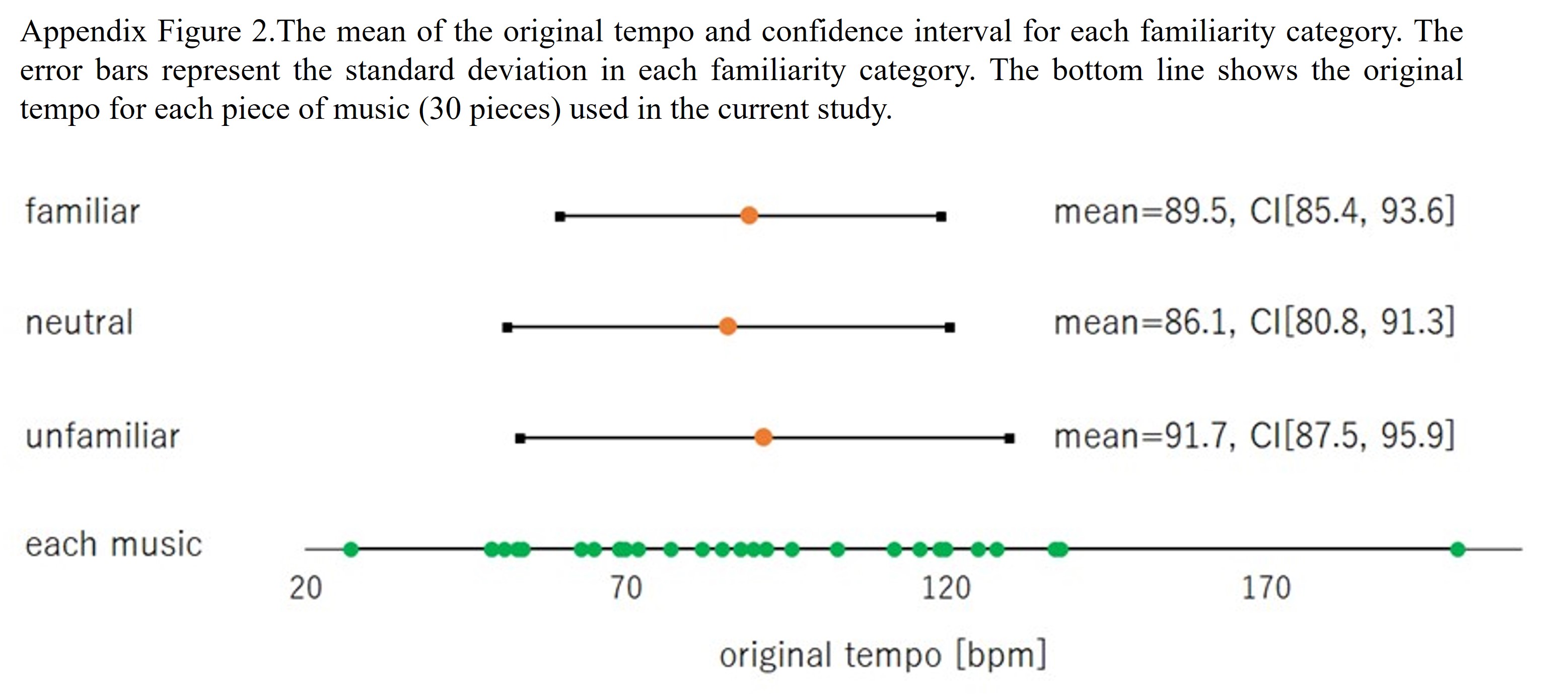

Supplement: Supplementary file 5 [file Image_2.jpg]
